# In Vitro Activity of New Cephalosporins vs *Streptococcus pneumoniae* from the Canadian Bacterial Surveillance Network: 2008–2011

**DOI:** 10.1007/s00284-014-0622-8

**Published:** 2014-07-15

**Authors:** Karen Green, Allison McGeer, Wallis Rudnick, Sylvia Pong-Porter, Samir N. Patel, Donald E. Low, The Canadian Bacterial Surveillance Network (CBSN)

**Affiliations:** 1 Department of Microbiology, Mount Sinai Hospital, Toronto, ON Canada; 2 Department of Laboratory Medicine and Pathobiology, University of Toronto, Toronto, ON Canada; 3 Ontario Agency for Health Protection and Promotion, Toronto, ON Canada

## Abstract

Between 2008 and 2011, 6,895 *Streptococcus pneumoniae* isolates were submitted to the Canadian Bacterial Surveillance Network and underwent in vitro susceptibility testing. Fifteen percent of *S. pneumoniae* isolates were collected from pediatric patients (0–15 years old), 48.6 % of isolates were collected from adults between 16 and 64 years of age, and 36.1 % from adults aged ≥65 years; age data were not available for 11 patients. Forty-five percent of *S. pneumoniae* isolates were recovered from sterile specimens, and 55 % of isolates were from nonsterile specimens. Overall, 0.4 % of isolates were resistant to penicillin, 0.4 % to ceftriaxone, 3 % to amoxicillin, 25 % to erythromycin, and 13 % to trimethoprim/sulfamethoxazole; 6.6 % of isolates were multidrug resistant (MDR). Among MDR isolates, resistance rates exceeded 95 % for erythromycin, tetracycline, and trimethoprim/sulfamethoxazole. The MIC_90_ of cethromycin, ceftaroline, and ceftobiprole against MDR isolates were 0.12, 0.25, and 1 mg/L, respectively. Ceftaroline, the active form of the prodrug ceftaroline fosamil, exhibited potent in vitro activity against the tested *S. pneumoniae* including all 456 multidrug-resistant strains. No ceftaroline-resistant isolates were identified.

## Introduction

*Streptococcus pneumoniae* is the most common bacterial pathogen associated with community-acquired bacterial pneumonia (CABP) [10, 14]. The use of pneumococcal conjugate vaccines has decreased the incidence of invasive pneumococcal disease. However, the number of strains that are resistant to commonly used antibiotics continues to increase [6].

Ceftaroline, the active form of the prodrug ceftaroline fosamil, is a parenteral cephalosporin exhibiting broad spectrum in vitro bactericidal activity against gram-positive pathogens, including multidrug-resistant (MDR) *S. pneumoniae* and methicillin-resistant *Staphylococcus aureus*, and common gram-negative organisms [5, 12, 15]. Ceftaroline fosamil is approved in the United States for the treatment of patients with CABP and acute bacterial skin and skin structure infections and for similar indications in Europe [16, 18]. We previously demonstrated that ceftaroline was the most active β-lactam agent tested against a subset of 260 MDR *S. pneumoniae* isolates collected across Canada between 2003 and 2008 [13].

The Canadian Bacterial Surveillance Network (CBSN) has collected *S. pneumoniae* isolates as part of a nationwide surveillance program since 1988. In recent years, there has been not only an increase in the prevalence of MDR *S. pneumoniae*, but also an increase in the degree of resistance to the β-lactam antibiotics. Surveillance studies in the United States also indicate an increase in nonsusceptibility of *S. pneumoniae* to common β-lactam antibiotics [6, 7]. The objective of this study was to assess the in vitro activity of ceftaroline and comparative agents against CBSN *S. pneumoniae* isolates collected from 2008 to 2011.

The CBSN encompasses volunteer community and hospital-affiliated laboratories across Canada, which provide services to community and tertiary-care hospitals, community clinics, physician offices, and long-term care facilities. All ten Canadian provinces and two of three territories are represented. In total, 186 laboratories have participated in the CBSN, with 40 laboratories submitting annually since 1993. Only one isolate per patient episode is included; laboratories are asked to submit all sterile-site isolates and a defined number of consecutive nonsterile-site isolates annually, based on laboratory size. All isolates are submitted to a central laboratory where they are confirmed as *S. pneumoniae* and serotyped using latex antisera (Statens Serum Institute, Denmark) and Quellung reaction [17]. Isolates that cannot be serotyped at the central laboratory are serotyped at Canada’s National Microbiology Laboratory. Broth microdilution susceptibility testing is performed and interpreted according to the Clinical and Laboratory Standards Institute (CLSI) guidelines [1]. For this study, nonmeningeal breakpoints for ceftaroline, penicillin, amoxicillin, and ceftriaxone are used to interpret MIC results [1]. In addition, an analysis by meningeal breakpoints was included to determine resistant isolates to penicillin and ceftriaxone.

From 2008 to 2011, 6,895 *S. pneumoniae* isolates from 59 centers underwent antimicrobial susceptibility testing. There were 1,043 (15.1 %) isolates collected from pediatric patients (0–15 years old), 3,350 (48.6 %) isolates collected from adults between 16 and 64 years of age, and 2,491 (36.1 %) from adults aged ≥65 years; age data were not available for 11 patients. Of 6,895 isolates, 3,088 (45 %) were recovered from sterile specimens (2,868 blood, 76 cerebral spinal fluid, 63 pleural fluid, and 81 other), and 3,796 (55 %) isolates were from nonsterile specimens (2,572 sputum, 417 eye, 247 ear, and 560 other).

Among sterile-site isolates, the most common serotypes were 19A (17 %), 7F (13 %), and 3 (8 %). Among nonsterile-site isolates, the most common serotypes isolated were 19A (11 %), 3 (9 %), and 11A (9 %). There were decreases in many common serotypes following the introduction of PCV10 in some provinces in 2009 and PCV13 in 2010. Serotypes included in PCV10 (1, 4, 5, 6B, 7F, 9 V, 14, 18C, 19F, 23F) and PCV13 (all in PCV10 and 3, 6A, 19A) accounted for 53.9 % of isolates in 2008, but this decreased to 44.6 % of isolates in 2011.

**Fig. 1:**
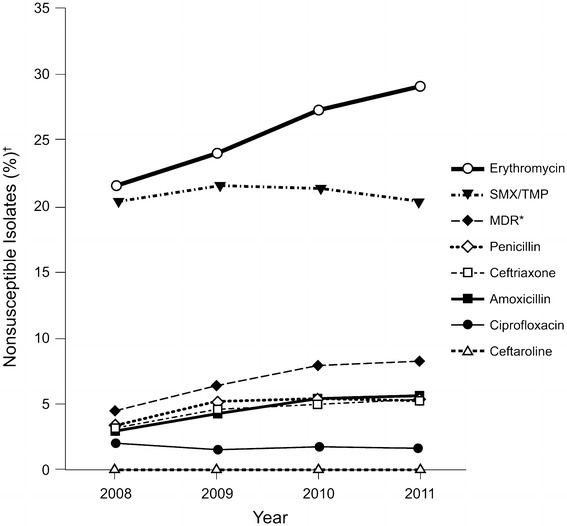
Percent of isolates nonsusceptible to common antibiotics and multidrug-resistant (MDR) isolates by year, 2008–2011. *MDR = multidrug-resistant, resistant to >2 classes of antibiotics (classes: β-lactams [penicillin/amoxicillin/ceftriaxone], erythromycin, tetracycline, trimethoprim/sulfamethoxazole, ciprofloxacin). ^†^Nonsusceptibility based on CLSI [17] interpretive breakpoints (amoxicillin MIC >2 mg/L; ceftaroline MIC >0.5 mg/L; ceftriaxone MIC >1 mg/L; erythromycin MIC >0.25 mg/L; penicillin MIC >2 mg/L; trimethoprim/sulfamethoxazole (SMX/TMP) MIC >0.5 mg/L) and MIC >2 mg/L for ciprofloxacin

The proportion of isolates resistant to more than two classes of antibiotics (MDR isolates) increased over time (Fig. 1). Overall, 6.6 % (456/6,895) of pneumococcal isolates were MDR. Among MDR isolates, resistance rates exceeded 95 % for erythromycin, tetracycline, and trimethoprim/sulfamethoxazole (Table 1). The MIC_90_ of cethromycin, ceftaroline, and ceftobiprole against MDR isolates were 0.12, 0.25, and 1 mg/L, respectively (Table 2). The highest MICs observed for ceftaroline, ceftobiprole, and cethromycin were 0.5, 2, and 4 mg/L, respectively.

**Table 1:** Percent of resistant *Streptococcus pneumoniae* isolates from Canada, 2008–2011

Drug	Percent (%) of isolates resistant	
	All isolates (*N* = 6895)	MDR^a^ isolates *(n* = 456) [*n*/*N* = 6.6 %]
Penicillin (nonmeningitis)	0.4	6.1
Penicillin (meningitis)	18.6	87.1
Amoxicillin^b^	3.4	51.1
Ceftriaxone (nonmeningitis)	0.4	6.1
Ceftriaxone (meningitis)	4.6	63.4
Erythromycin^c^	25.1	99.8
High-level	13.2	82.5
Low-level	12.0	17.3
Trimethoprim/sulfamethoxazole	12.7	98.5
Tetracycline	12.9	96.7
Ciprofloxacin	1.8	8.1

^a^ *MDR* multidrug-resistant, resistant to >2 classes of antibiotics (classes: β-lactams [penicillin/amoxicillin/ceftriaxone], erythromycin, tetracycline, trimethoprim/sulfamethoxazole, ciprofloxacin)

^b^Nonmeningeal breakpoints used

^c^High-level erythromycin resistance = MIC ≥16 mg/L; low-level erythromycin resistance = MIC ≥1 to <16 mg/L

**Table 2:**
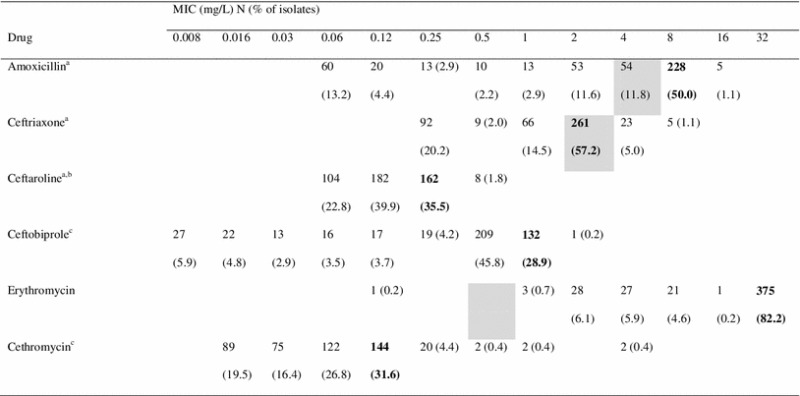
In vitro activities of antimicrobial agents against multidrug-resistant *Streptococcus pneumoniae* isolates from Canada, 2008–2011 (N = 456)

Bolded values = MIC_90_; gray boxes = intermediate MIC values as defined by CLSI

^a^Nonmeningeal breakpoints

^b^MIC susceptibility breakpoint: ≤0.5 mg/L

^c^MIC breakpoints not established by CLSI

The MIC_90_ of ceftaroline was ≥8-fold lower, the MIC_90_ of ceftobiprole was ≥2-fold lower, and the MIC_90_ of cethromycin was ≥16-fold lower than the MIC_90_ of ceftriaxone across penicillin-, amoxicillin-, or erythromycin-resistant isolates and in MDR isolates (Table 3). The MIC_90_ of ceftriaxone increased over the study period from 0.25 mg/L in 2008 to 0.5 mg/L in 2011 (data not shown). Additionally, among all isolates, 13.2 % demonstrated high-level erythromycin resistance (MIC ≥16 mg/L) and 12.0 % demonstrated low-level erythromycin resistance (MIC ≥1 to <16 mg/L) (Table 1).

**Table 3:** In vitro activities of antimicrobial agents against *Streptococcus pneumoniae* isolates from Canada, 2008–2011

MIC	Ceftriaxone		Ceftaroline		Ceftobiprole		Cethromycin	
(mg/L) (*N*)	MIC_50_	MIC_90_	MIC_50_	MIC_90_	MIC_50_	MIC_90_	MIC_50_	MIC_90_
Penicillin								
<8 (6867)	0.25	0.25	0.06	0.06	0.016	0.25	0.016	0.06
≥8 (28)	4	8	0.25	0.5	1	1	0.12	0.25
Amoxicillin								
<8 (6660)	0.25	0.25	0.06	0.06	0.016	0.06	0.016	0.03
≥8 (235)	2	4	0.25	0.25	0.5	1	0.12	0.12
Erythromycin								
< 1 (5164)	0.25	0.25	0.06	0.06	0.016	0.03	0.016	0.016
≥1 (1731)	0.25	2	0.06	0.25	0.03	0.5	0.03	0.12
MDR								
No (6439)	0.25	0.25	0.06	0.06	0.016	0.06	0.016	0.03
Yes (456)	2	2	0.12	0.25	0.5	1	0.06	0.12

MDR = multidrug-resistant, resistant to > 2 classes of antibiotics (classes: β-lactams [penicillin/amoxicillin/ceftriaxone], erythromycin, tetracycline, trimethoprim/sulfamethoxazole, ciprofloxacin)

Emerging *S. pneumoniae* resistance, particularly for macrolides, is evident based on these surveillance data and reports from SENTRY [6]. High-level macrolide resistance is increasing, with more than half of erythromycin-resistant isolates considered to have high-level resistance in this study. Guidelines may no longer be able to recommend macrolides for first-line therapy based on >25 % resistance levels [9]. Resistance to β-lactam agents, apart from ceftaroline, also increased throughout the study period.

Ceftaroline, ceftobiprole, and cethromycin exhibited more potent in vitro activity against MDR pneumococci than ceftriaxone. Potent in vitro activity of ceftaroline against pneumococci has also been reported from the Assessing Worldwide Antimicrobial Resistance Evaluation (AWARE) program [4]. In vitro activity of ceftaroline can be attributed to its high affinity for *S. pneumoniae* penicillin-binding proteins (PBPs), including PBPs 1a, 2b, and 2x [8, 11]. In an integrated analysis of 2 phase 3 clinical trials comparing ceftaroline fosamil with ceftriaxone in the treatment of patients with CABP, clinical cure at the test-of-cure visit was higher in the ceftaroline fosamil group than in the ceftriaxone group in patients with *S.* *pneumoniae* (85.5 vs 68.6 %, respectively) [3]. An analysis of patients in these trials that evaluated clinical response rates at an earlier end point, 72 h after initiation of therapy, showed similar results, with 73 % of patients in the ceftaroline fosamil group compared with 56 % of patients in the ceftriaxone group experiencing clinical response following a *S. pneumoniae* infection (*P* = 0.03) [2].

In summary, the percentage of MDR *S. pneumoniae* isolates increased from approximately 5 % in 2008 to 8 % in 2011. Among the β-lactam antibiotics tested, ceftaroline demonstrated the most potent in vitro activity against MDR *S. pneumoniae*. The highest MIC observed for ceftaroline against any *S. pneumoniae* isolate was 0.5 mg/L. These data suggest that ceftaroline fosamil can play an important role in the treatment of infection caused by *S. pneumoniae*, including MDR strains. Based on the high clinical and microbiological response rates in clinical trials and the potent in vitro activity against *S. pneumoniae* in this analysis, ceftaroline fosamil is a useful option for management of CABP.

## Appendix

L.P. Abbott, Dr. Everett Chalmers Regional Hospital, Fredericton, New Brunswick; H. Almohri, Lifelabs Medical Laboratory Services, Ontario; M. Alfa, St. Boniface General Hospital, Winnipeg, Manitoba; A. Belhaj, Rouge Valley Health System, Toronto, Ontario; J. Blondeau, Royal University Hospital, Saskatoon, Saskatchewan; L. Bocci, Chaleur Regional Hospital, Bathurst, New Brunswick; W. Ciccotelli, Grand River Hospital, Kitchener, Ontario; R. Davidson and K. Forward, QEII Elizabeth Health Sciences Centre, Halifax, Nova Scotia; H.R. Devlin, St. Michael’s Hospital, Toronto, Ontario; J. Downey, Toronto East General Hospital, Toronto, Ontario; S. El-Bailey, Saint John Regional Hospital, Saint John, New Brunswick; G. German, Queen Elizabeth Hospital, Charlottetown, Prince Edward Island; H. Giang, Ross Memorial Hospital, Lindsay, Ontario; D. Hoban and G. Zhanel, Health Sciences Centre, Winnipeg, Manitoba; Y. Hussein, Cape Breton Regional Hospital, Sydney, Nova Scotia; K. Katz, North York General Hospital, North York, Ontario and Shared Hospital Laboratory Inc, Toronto, Ontario; P.C. Kibsey, Royal Jubilee Hospital, Victoria, British Columbia and Nanaimo District General Hospital, Nanaimo, British Columbia; S. Krajden, St. Joseph’s Health Centre, Toronto, Ontario; M. Kuhn, The Moncton Hospital, Moncton, New Brunswick; P.R. Laberge, Centre Hospitalier Regional de Sept-Iles, Sept-Iles, Quebec; K.S. Lee, Humber River Regional Hospital, Toronto, Ontario; B. Nash, Whitehorse General Hospital, Whitehorse, Yukon; D. Noria, The Scarborough Hospital, Toronto, Ontario; K. Ostrowska and A. Sarabia, Trillium Health Partners, Toronto, Ontario; P. Pieroni, Westman Regional Laboratory, Brandon, Manitoba; R. Price, Royal Victoria Hospital, Barrie, Ontario; N. Rau, Halton Healthcare, Oakville, Ontario; D. Richardson, William Osler Health Services, Brampton, Ontario and Headwaters Health Care Centre, Orangeville, Ontario; S. Richardson, Hospital for Sick Children, Toronto, Ontario; V. Sales, Markham Stouffville Hospital, Markham, Ontario; A.E. Simor, Sunnybrook Health Sciences Centre, Toronto and Lakeridge Health, Oshawa, Ontario; G. Tyrrell, Alberta Provincial Health Laboratory, Edmonton, Alberta.

## References

[1] Clinical and Laboratory Standards Institute (2013) M100-S23. Performance standards for antimicrobial susceptibility testing: 23rd informational supplement. Clinical and Laboratory Standards Institute, Wayne, PA

[2] Eckburg PB, Friedland HD, Llorens L, Smith A, Witherell GW, Laudano JB, Thye D (2012). Day 4 clinical response of ceftaroline fosamil versus ceftriaxone for community-acquired bacterial pneumonia. Infect Dis Clin Pract.

[3] File TM, Low DE, Eckburg PB, Talbot GH, Friedland HD, Lee J, Llorens L, Critchley I, Thye D (2010). Integrated analysis of FOCUS 1 and FOCUS 2: randomized, double-blinded, multicenter phase 3 trials of the efficacy and safety of ceftaroline fosamil versus ceftriaxone in patients with community-acquired pneumonia. Clin Infect Dis.

[4] Flamm RK, Sader HS, Farrell DJ, Jones RN (2012). Summary of ceftaroline activity against pathogens in the United States, 2010: report from the Assessing Worldwide Antimicrobial Resistance Evaluation (AWARE) Surveillance Program. Antimicrob Agents Chemother.

[5] Ge Y, Biek D, Talbot GH, Sahm DF (2008). In vitro profiling of ceftaroline against a collection of recent bacterial clinical isolates from across the United States. Antimicrob Agents Chemother.

[6] Jones RN, Sader HS, Mendes RE, Flamm RK (2013). Update on antimicrobial susceptibility trends among *Streptococcus pneumoniae* in the United States: report of ceftaroline activity from the SENTRY Antimicrobial Surveillance Program (1998–2011). Diagn Microbiol Infect Dis.

[7] Jones RN, Sader HS, Moet GJ, Farrell DJ (2010). Declining antimicrobial susceptibility of *Streptococcus pneumoniae* in the United States: report from the SENTRY Antimicrobial Surveillance Program (1998–2009) [letter]. Diagn Microbiol Infect Dis.

[8] Kosowska-Shick K, McGhee PL, Appelbaum PC (2010). Affinity of ceftaroline and other β-lactams for penicillin-binding proteins from *Staphylococcus aureus* and *Streptococcus pneumoniae*. Antimicrob Agents Chemother.

[9] Low DE (2013). What is the relevance of antimicrobial resistance on the outcome of community-acquired pneumonia caused by *Streptococcus pneumoniae*? (Should macrolide monotherapy be used for mild pneumonia?). Infect Dis Clin North Am.

[10] Mandell LA, Wunderink RG, Anzueto A, Bartlett JG, Campbell GD, Dean NC, Dowell SF, File TM, Musher DM, Niederman MS, Torres A, Whitney CG (2007). Infectious Diseases Society of America/American Thoracic Society consensus guidelines on the management of community-acquired pneumonia in adults. Clin Infect Dis.

[11] Moisan H, Pruneau M, Malouin F (2010). Binding of ceftaroline to penicillin-binding proteins of *Staphylococcus aureus* and *Streptococcus pneumoniae*. J Antimicrob Chemother.

[12] Mushtaq S, Warner M, Ge Y, Kaniga K, Livermore DM (2007). In vitro activity of ceftaroline (PPI-0903 M, T-91825) against bacteria with defined resistance mechanisms and phenotypes. J Antimicrob Chemother.

[13] Patel SN, Pillai DR, Pong-Porter S, McGeer A, Green K, Low DE (2009). In vitro activity of ceftaroline, ceftobiprole and cethromycin against clinical isolates of *Streptococcus pneumoniae* collected from across Canada between 2003 and 2008 [letter]. J Antimicrob Chemother.

[14] Richter SS, Heilmann KP, Dohrn CL, Riahi F, Beekmann SE, Doern GV (2009). Changing epidemiology of antimicrobial-resistant *Streptococcus pneumoniae* in the United States, 2004–2005. Clin Infect Dis.

[15] Sader HS, Fritsche TR, Kaniga K, Ge Y, Jones RN (2005). Antimicrobial activity and spectrum of PPI-0903 M (T-91825), a novel cephalosporin, tested against a worldwide collection of clinical strains. Antimicrob Agents Chemother.

[16] Teflaro^®^ (ceftaroline fosamil) [prescribing information] (2012) Forest Pharmaceuticals, Inc., St. Louis, MO

[17] World Health Organization (2003) Manual for the laboratory identification and antimicrobial susceptibility testing of bacterial pathogens of public health concern in the developing world. Appendix 6. Serotyping and Quellung Typing of Streptococcus pneumoniae. p 255–258. http://www.who.int/csr/resources/publications/drugresist/WHO_CDS_CSR_RMD_2003_6/en/

[18] Zinforo™ summary of product characteristics (2012) AstraZeneca AB, Sodertalje, Sweden

